# Metabolic profiles in gestational diabetes mellitus can reveal novel biomarkers for prediction of adverse neonatal outcomes

**DOI:** 10.3389/fped.2024.1432113

**Published:** 2024-08-21

**Authors:** Xiaoxiao Yin, Tingting Yu, Dongmei Jiang, Chunjian Shan, Jiaai Xia, Min Su, Min Zhang, Ling Chen, Hong Zhong, Xianwei Cui, Chenbo Ji

**Affiliations:** ^1^ Women's Hospital of Nanjing Medical University, Nanjing Women and Children's Healthcare Hospital, Nanjing, Jiangsu, China; ^2^ School of Nursing, Nanjing Medical University, Nanjing, Jiangsu, China; ^3^ Department of Obstetrics and Gynecology, Affiliated Hospital of Nantong University, Nantong, China

**Keywords:** GDM, hypoglycemia, macrosomia, metabolomics, prediction biomarkers

## Abstract

**Background:**

Gestational diabetes mellitus (GDM) significantly affects the fetal metabolic environment, elevating risks of neonatal hypoglycemia and macrosomia. Metabolomics offers promising avenues for early prediction and diagnosis of GDM and associated adverse offspring outcomes.

**Methods:**

This study analyzed serum samples from pregnant women diagnosed with GDM at 24 to 28 weeks of gestation using untargeted metabolomics. We monitored the health outcomes of their offspring to explore the correlation between initial serum metabolite profiles and subsequent health outcomes, to uncover the predictive markers for hypoglycemia and macrosomia in these offspring.

**Results:**

Out of 200 participants, 154 had normal newborns, 33 had offspring with hypoglycemia, and 19 had offspring with macrosomia. From 448 identified metabolites, 66 showed significant differences in cases of hypoglycemia, and 45 in macrosomia. A panel of serum metabolite biomarkers achieved Area Under the Curve (AUC) values of 0.8712 for predicting hypoglycemia and 0.9434 for macrosomia.

**Conclusion:**

The study delineated metabolic disruptions in GDM during 24–28 weeks of gestation and pinpointed biomarkers capable of forecasting adverse neonatal outcomes. These findings could inform GDM management strategies and minimize the incidence of such outcomes.

## 1 Introduction

Gestational diabetes mellitus (GDM), which is characterized by variations in glucose tolerance that first appear or are observed during pregnancy (1), accounts for 80%–90% of the cases of pregnancy-related hyperglycemia. A meta-analysis show that the global GDM prevalence was 14.2% by 2021 according to the IADPSG diagnostic criteria (2). The incidence of adverse pregnancy outcomes such as hypoglycemia, macrosomia, and hyperbilirubinemia in the offspring of women with GDM has been reported to be 1.16–2.02-fold higher than that in cases with normal glucose hemostasis (3, 4). Therefore, preventing or treating GDM and avoiding the occurrence of adverse pregnancy outcomes is clinically important.

Despite diet and exercise management during pregnancy, GDM is associated with a high risk of neonatal hypoglycemia and macrosomia. Ambient hyperinsulinemia plays a crucial role in the development of fetal macrosomia and neonatal hypoglycemia (5). In severe cases, neonates with hypoglycemia may show feeding difficulties and brain damage, resulting in intellectual disability and permanent nerve damage (6). Similarly, macrosomia affects maternal and perinatal health and increases the risk of metabolic diseases such as obesity, diabetes, and hyperlipidemia in adulthood (7). Numerous studies have demonstrated that elevated fasting blood glucose (FBG) (8), glycated hemoglobin (HbA1c) (9), triglyceride (TG), total cholesterol (TC), low-density lipoprotein cholesterol (LDL-C), and high-density lipoprotein cholesterol (HDL-C) (10) levels are associated with adverse maternal and infant outcomes in cases of GDM. However, these clinical indicators are not sufficient to predict the risk of neonatal hypoglycemia and macrosomia.

Metabolomics has been recently used to predict and diagnose GDM (11, 12). The changes in metabolites directly reflect the activities or processes that have occurred or are currently occurring within an organism (13). Untargeted metabolomics can provide insights into the role of metabolites in physiological and pathological conditions (14, 15). Serum metabolomic studies have identified significant changes in metabolites such as iconic acid, glucosamine, and tetrahydrocortisone, making them potential biomarkers for early GDM diagnosis (16–18). Metabolomics has also been used to predict the risk of progression from GDM to type 2 diabetes mellitus in the early postpartum period (19), indicating that metabolite disorders occur before the diagnosis or progression of GDM and that analysis of the metabolic spectrum is a practical approach to discovering early biomarkers. Importantly, since GDM leads to metabolic disorders in women and affects the fetal metabolic environment, untargeted metabolomics is a useful technique for identifying early-stage metabolite predictors to avoid adverse neonatal outcomes in cases of GDM.

Therefore, in this study, we conducted untargeted metabolomics analyses using pregnancy samples of patients with GDM at 24 to 28 weeks and followed-up the neonatal outcomes to investigate the metabolic changes in cases of GDM with adverse neonatal outcomes and to predict the risk of neonatal hypoglycemia and macrosomia using serum metabolites.

## 2 Materials and methods

### 2.1 Study population and study design

For this study, we recruited women diagnosed with GDM based on the IADPSG criteria (International Association of Diabetes and Pregnancy Study Groups Consensus Panel et al.) at Nanjing Women and Children's Healthcare Hospital in 2022. According to the IADPSG criteria, GDM is diagnosed by FBG level ≥ 5.1 mmol/L, 1-h postprandial glucose level ≥ 10.0 mmol/L, or 2-h postprandial glucose level ≥ 8.5 mmol/L. The study included mothers aged 25–35 years who had natural and singleton pregnancies and underwent a 2-h 75-g oral glucose tolerance test (OGTT) between 24 and 28 weeks of gestation. Their pre-pregnancy body mass index (BMI) ranged from 18.5 to 28 kg/m^2^. The study participants were all Chinese. Mothers with abnormal glucose metabolism or diabetes mellitus before pregnancy, neurological dysfunction, cognitive disorders, cardiac issues, malignant tumors, pulmonary failure, renal diseases, or any other diseases were excluded. The study protocol was established in accordance with the ethical guidelines of the Helsinki Declaration and was approved by the Human Ethics Committee of Nanjing Women and Children's Healthcare Hospital (No: 2020KY-075). Participants volunteered to take part in the study and provided informed consent. Fasting serum samples were collected at the time of the OGTT and stored at −80°C for the subsequent metabolomics studies.

The participants' clinical data were also collected from 24 to 28 weeks of gestation, and their pregnancy outcomes were followed-up. Data for TC, TG, and HbA1c levels were missing in the group with no neonatal abnormalities, and the missing data rate was 0.5%. We adopted a multiple interpolation approach to impute the missing data. The study design is shown in Supplementary Figure S1. The study cohort was divided into three groups on the basis of the pregnancy outcomes. The Case N group included all cases with no neonatal abnormalities; the Case A group included cases showing neonatal hypoglycemia (blood glucose < 2.2 mmol/L within 48 h of birth); and the Case B group included cases showing neonatal macrosomia (birth weight ≥ 4,000 g). Patients who could not be traced due to various reasons and cases involving other neonatal diseases were excluded.

For creating the predictive models, we used shrinkage methods to estimate the sample size. Shrinkage methods deal with the problem of overfitting by reducing the variability in the developed model's predictions such that extreme prediction (20). The formula is as follows, where *n* is the sample size, *P* = 5 (number of alternative predictor variables), S = 0.9 (shrinkage factor), and *R*^2^CS = 0.2 (Cox-Snell *R*^2^, a conservative metric for evaluating the performance of the model). This approach indicated that a sample size of 198 cases was required for this study.

$$n=\frac{P}{(S-1)\ln\left(1-\frac{R_{\mathrm{CS}}^{2}}{S}\right)}$$

Finally, 200 samples were collected and were sufficient to meet the sample size requirements and biological replication for untargeted metabolomics analysis (21–23).

### 2.2 Untargeted metabolomics analysis

#### 2.2.1 Detection and identification of serum metabolites

Untargeted metabolomics analysis was conducted using a high-performance liquid chromatography-mass spectrometry (HPLC-MS) unit (Biotree Biomedical Technology Company, Shanghai). Quality control samples were prepared by pooling all samples to evaluate the stability of subsequent tests. To avoid systematic error, deviation values were filtered, and metabolite data with ≥50% missing values in a single group and ≥50% missing values in all groups were excluded. Then, the remaining missing values were filled by multiplying the minimum value by a random number between 0.1 and 0.5. Finally, metabolite identification was achieved through a spectral match using the Human Metabolome Database (HMDB) and Kyoto Encyclopedia of Genes and Genomes (KEGG).

#### 2.2.2 Data processing

First, principal component analysis (PCA), an unsupervised analysis, was used to visualize the distribution and grouping of the samples. We generated all PCA plots using SIMCA software, with ellipses marking the 95% confidence intervals used to identify potential outliers in the dataset. Second, orthogonal projections to latent structures-discriminant analysis (OPLS-DA) was used to reflect the differences between groups and discriminate significantly changed metabolites. Finally, the value for the variable importance in the projection (VIP) of the first principal component in the OPLS-DA analysis was acquired to summarize the contribution of each variable to the model. Metabolites with VIP > 1 (by OPLS-DA) and *P* < 0.05 (by Student's *t*-test) were considered to show significant changes (24).

### 2.3 Pathway analysis

A public database was used for pathway enrichment analysis with KEGG and MetaboAnalyst 5.0. Based on the enrichment results of the differential metabolites in KEGG metabolic pathways, the differential abundance score was obtained by calculating the ratio of the difference between the number of annotated upregulated differential metabolites and the number of downregulated differential metabolites in a specific pathway to the number of all metabolites in this pathway, which could reflect the overall change of all the different metabolites in a pathway. To further screen the pathways and find the critical pathways showing the highest correlation with the differential metabolites, we performed enrichment and topological analyses of these pathways.

### 2.4 Predictive analytics

The sample for this study was an unbalanced dataset. We used the Synthetic Minority Oversampling Technique (SMOTE) to balance it (25). SMOTE was performed using the “UBL” package. New samples were added to the dataset by synthesizing them artificially based on k nearest-neighbor sampling with the value of k set to 5. The sample size of the dataset for the Case A (hypoglycemia) and Case B (macrosomia) groups was increased to 151 and 152 cases respectively. In this study, the “randomForest” package was used to analyze the sample dataset. The random forest (RF) algorithm is a state-of-the-art machine learning method used to develop predictive models. It can be used for predictor variables of various sizes or distributions and is suitable for application in high-dimensional environments where the number of predictor variables may be greater than the number of observations. In addition, this method can emphasize the relevance of each predictor variable through the use of so-called variable significance measures. Therefore, this method is well-suited for analyzing complex data, such as omics data (26). The dataset was divided into training and validation sets in a 7:3 ratio through random sampling. The mean Gini index reduction in clinical factors and differential metabolites was calculated to identify the five variables that had the most significant impact on adverse maternal and infant outcomes in patients with GDM. These five variables were then used to construct a risk-prediction model. The performance of the prediction model was evaluated using the area under the receiver operating characteristic curve (AUC) metric. Both of the above packages are from RStudio 4.2.2.

## 3 Results

### 3.1 Clinical characteristics of the participants

This study enrolled 200 pregnant women with GDM at 24–28 weeks of gestation, including 154 cases with no neonatal abnormalities (Case N), 33 cases of neonatal hypoglycemia (Case A), and 19 cases of neonatal macrosomia (Case B). The clinical characteristics are summarized in Table 1. The three groups showed no significant differences in prenatal and sociodemographic characteristics (age, BMI, gravidity, and parity). The clinical indicators in the Case A group (gestational week in OGTT, systolic blood pressure, diastolic blood pressure, and FBG, 2-h blood glucose, HDL-C, LDL-C, TG, and TC levels) showed no significant differences from those in the Case N group. However, at 24–28 weeks of gestation, the HbA1c level in the Case B group was considerably higher than that in the Case N group (*P* = 0.005), but it was still within the clinical normal reference range. Each of these 14 clinical characteristics were included in the subsequent predictive analyses. In the assessment of pregnancy outcomes, neonates from the Case A and Case B groups showed significantly lower blood glucose levels (*P* < 0.001, *P* = 0.005) and higher birth weights (*P* < 0.001, *P* < 0.001), respectively, than the corresponding values in the Case N group. In terms of the sex of the neonates, the number of males was slightly higher than that of females in the Case N group; the male/female ratio was balanced in the Case A group; and the number of male neonates was significantly greater than that of female neonates in the Case B group. Baseline data showed no significant differences in clinical characteristics between GDM patients with and without neonatal adverse outcomes at 24–28 weeks of gestation, which suggested that only focusing on clinical indicators could not predict the occurrence of adverse neonatal outcomes.

**Table 1:** Clinical characteristics of pregnant women with GDM.

Clinical characteristics	Case N	Case A	Case B	Case N vs. Case A *P*-values	Case N vs. Case B *P*-values
Prenatal and sociodemographic characteristics					
Age (years)	29.42 ± 2.79	29.36 ± 2.56	29.47 ± 2.97	0.912	0.940
Pre-pregnancy BMI (kg/m^2^)	20.94 ± 1.84	21.12 ± 1.66	21.52 ± 1.36	0.611	0.188
Gravidity, *n*	1.62 ± 0.86	1.58 ± 1.15	1.84 ± 1.26	0.816	0.312
Parity, *n*	0.29 ± 0.49	0.27 ± 0.45	0.32 ± 0.58	0.89	0.807
Clinical indicators at 24–28 weeks of gestation					
Gestational week(OGTT)	25.90 ± 0.73	25.86 ± 0.65	25.87 ± 0.70	0.773	0.889
SBP (mmHg)	108.62 ± 9.96	107.48 ± 11.31	111.21 ± 11.02	0.562	0.293
DBP (mmHg)	69.37 ± 7.66	69.55 ± 8.64	68.47 ± 8.04	0.907	0.633
FBG (mmol L ^−1^)	4.62 ± 0.41	4.57 ± 0.36	4.74 ± 0.43	0.457	0.245
2hBG (mmol L ^−1^)	8.67 ± 1.17	8.97 ± 1.19	8.29 ± 1.19	0.188	0.180
HbA1c, %	4.99 ± 0.20	4.98 ± 0.26	5.13 ± 0.27^b^	0.985	0.005
HDL-C (mmol/L)	2.30 ± 0.37	2.34 ± 0.42	2.14 ± 0.34	0.620	0.075
LDL-C (mmol/L)	2.92 ± 0.59	2.93 ± 0.72	3.01 ± 0.78	0.942	0.542
TG (mmol/L)	2.14 ± 0.66	2.14 ± 0.64	2.31 ± 0.62	0.995	0.280
TC (mmol/L)	5.98 ± 0.82	6.02 ± 0.94	5.98 ± 1.15	0.815	0.988
Offspring outcome index					
Blood glucose (mmol/L)	3.48 ± 0.89	1.90 ± 0.40^a^	2.87 ± 0.80^b^	<0.001	<0.001
Birth weight (kg)	3.27 ± 0.31	3.57 ± 0.42^a^	4.18 ± 0.22^b^	<0.001	<0.001
Sex					
Male	89 (57.79%)	16 (48.48%)	13 (68.42%)	–	–
Female	65 (42.21%)	17 (51.52%)	6 (31.58%)	–	–

Data are presented as mean (SD) or *n* (%). Student's *t*-test was used for continuous variables (mean, SD). The *P*-values corrected by Bonferroni correction. Case A, hypoglycemia; Case B, macrosomia. SBP, systolic blood pressure; DBP, diastolic blood pressure; FBG, fasting blood glucose; 2hBG, 2 h value of OGTT; HbA1c, glycosylated hemoglobin; HDL-C, high density lipoprotein; LDL-C, low density lipoprotein; TG, triglyceride; TC cholesterol.

^a^*P*-values <0.0167 for Case A vs. Control.

^b^*P*-values <0.0167 for Case B vs. Control.

### 3.2 Serum metabolomics profile of GDM with adverse pregnancy outcomes

**Figure 1:**
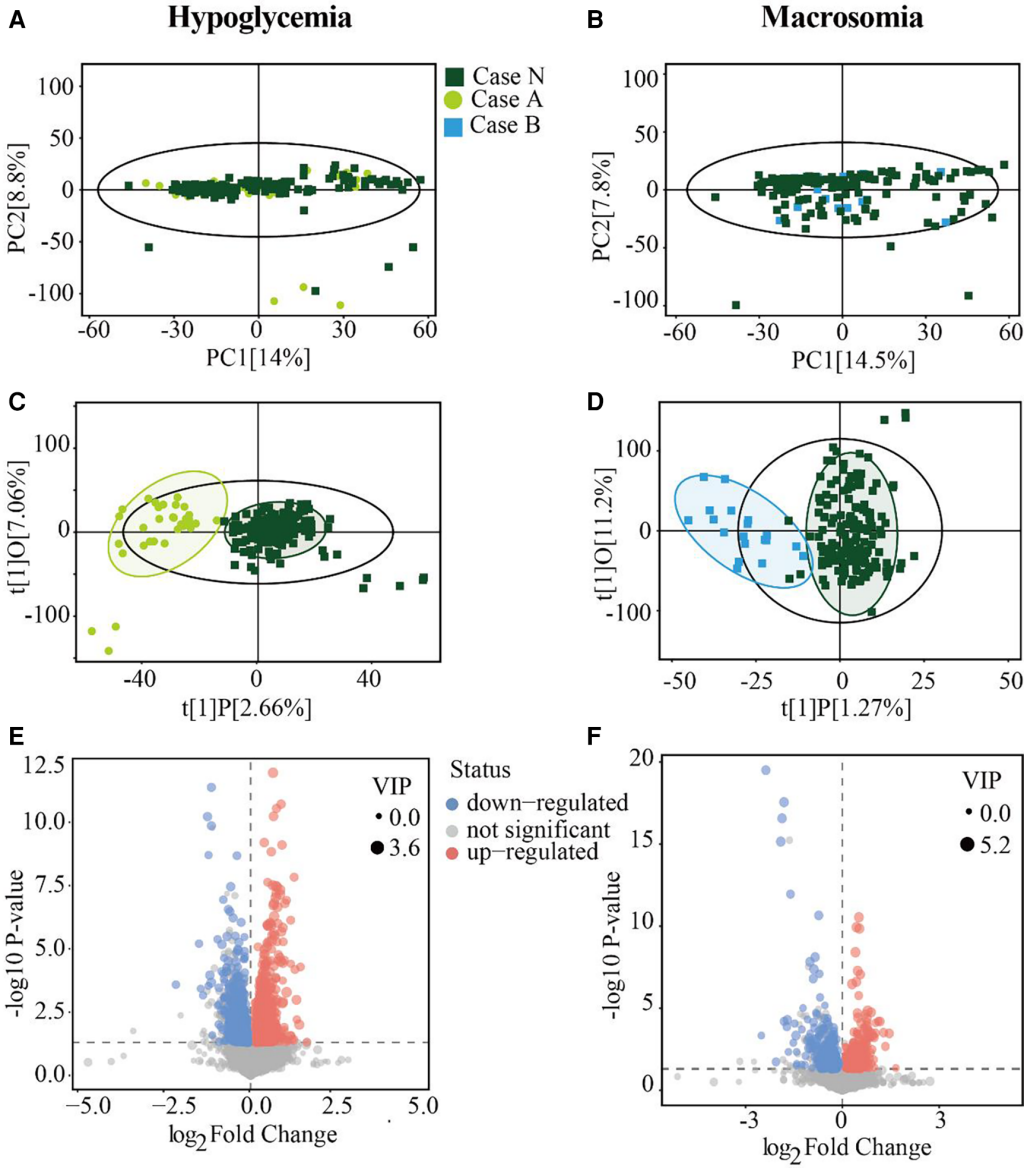
Serum metabolites differ between the GDM and case groups. **(A,B)** PCA score plots for Case N vs. Case A groups and Case N vs. Case B groups. **(C,D)** Score scatter plots of the OPLS-DA model for Case N vs. Case A groups and Case N vs. Case B groups respectively. **(E** and **F)** Volcano plot showing -log 10 (*P*-value) vs. log 2 (fold-change) for all metabolites. Areas of 95% confidence were highlighted in circles. PCA, principal component analysis; OPLS-DA, orthogonal projections to latent structures-discriminate analysis.

To explore the metabolite variations in pregnancy that corresponded to adverse pregnancy outcomes, we performed untargeted metabolomics analysis using serum samples obtained at 24–28 weeks of gestation from mothers with GDM in the Case N, A, and B groups. PCA and OPLS-DA analysis were used to summarize the variations and visualize the distribution in all samples. The PCA model showed that most samples were placed inside the 95% confidence interval (Figures 1A,B), whereas a supervised OPLS-DA analysis visibly distinguished the Case A and Case B groups from the Case N group (Figures 1C,D), indicating significant differences in the metabolomics profiles of GDM cases showing adverse pregnancy outcomes. Moreover, all groups were well-clustered, indicating that the differences in metabolic profiles between the Case N group and the Case A and Case B groups were significant.

A total of 26,661 peaks were obtained, and 21,874 peaks were retained after preprocessing and eliminating invalid data (Supplementary Table S1). On the basis of the VIP values calculated by OPLS-DA (VIP > 1) and Student's *t*-test (*P* < 0.05), Case A and Case B groups showed 2,696 and 760 changed features in comparison with the Case N group, respectively (Supplementary Table S2). Specifically, as shown in the volcano diagram, 1,964 features were upregulated and 732 features were downregulated in the Case A group (Figure 1E), while 317 features were upregulated and 443 features were downregulated in the Case B group (Figure 1F). These results together indicated that the metabolite expression patterns of GDM patients at 24–28 weeks of gestation showed significant changes before the occurrence of neonatal hypoglycemia or macrosomia.

### 3.3 Classification of metabolite changes in GDM with neonatal hypoglycemia or macrosomia

**Figure 2:**
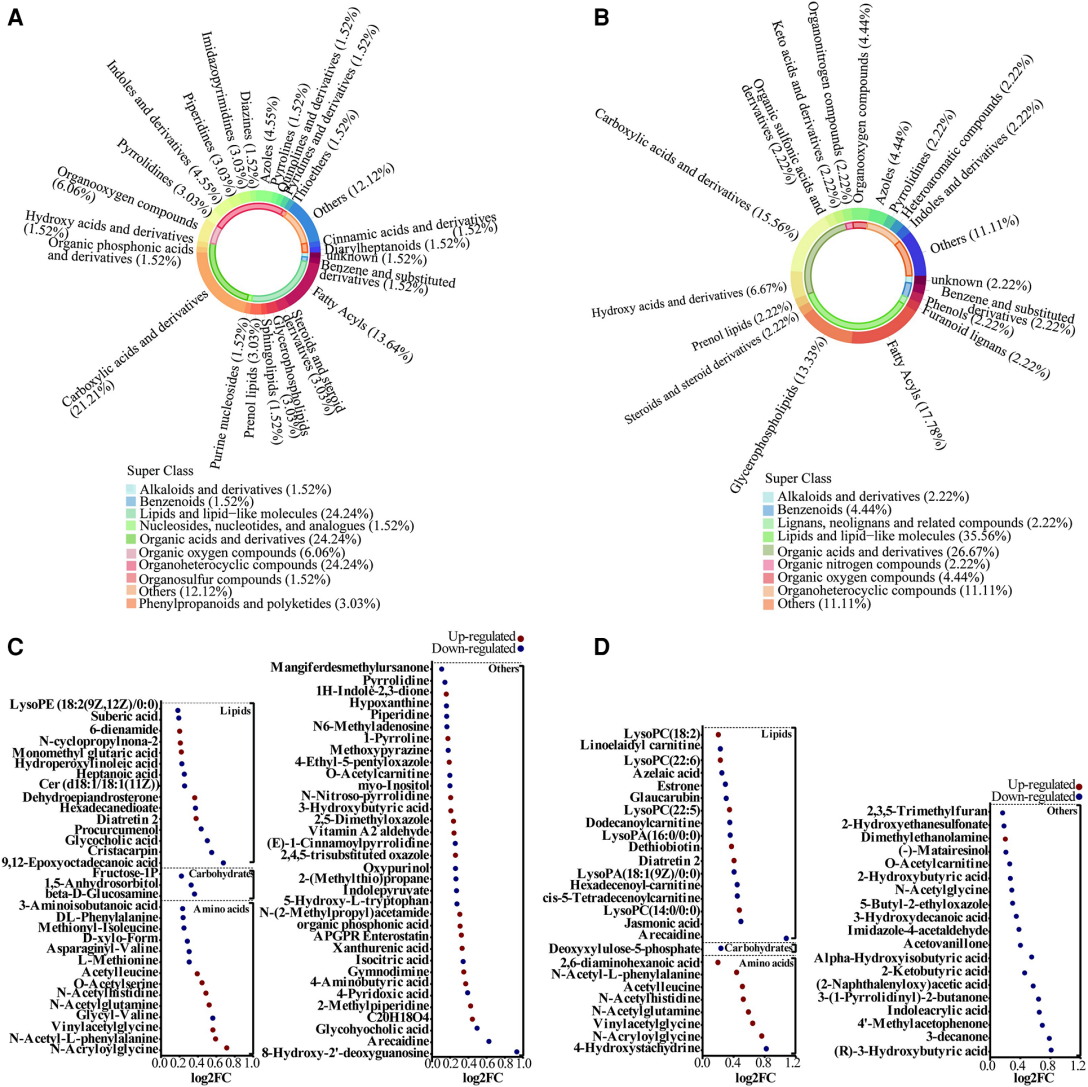
Alterations in metabolites species associated with adverse offspring outcomes. **(A,B)** Metabolite classification for Case A and Case B groups. **(C,D)** Bubble plot showing the differentially altered metabolites in Case A and Case B groups. Red indicates up-regulation and blue denotes down-regulation.

After matching with the Human Metabolome Database (HMDB), a total of 448 metabolites were identified. The secondary and tertiary classifications of the metabolites identified in the Case A group were shown in Figure 2A; the metabolites identified were mainly lipids and lipid-like molecules (24.24%), organic acids and derivatives (24.24%), and organoheterocyclic compounds (24.24%). The metabolites identified in the Case B group were mainly lipids and lipid-like molecules (35.56%) and organic acids and derivatives (26.67%), similar to the Case A group (Figure 2B). Among these metabolites, 66 were differential metabolites in Case A, of which 29 were upregulated and 37 were downregulated in comparison with the Case N group. Among the differential metabolites screened, carbohydrate metabolites were all downregulated (Figure 2C). We further screened 45 differentially expressed metabolites in the Case B group, of which 14 metabolites were upregulated and 31 were downregulated, with lipids showing a downward trend. In contrast, amino acids showed an upward trend (Figure 2D). Among these differentially expressed metabolites, eight were altered in both Case A and Case B groups, including Acetylleucine (VIP = 2.348, *P* < 0.001; VIP = 3.983, *P* < 0.001), Arecaidine (VIP = 1.702, *P* = 0.024; VIP = 1.193, *P* < 0.001), Diatretin 2 (VIP = 2.346, *P* < 0.001; VIP = 4.129, *P* < 0.001), N-Acetylglutamine (VIP = 2.399, *P* < 0.001; VIP = 4.010, *P* < 0.001), N-Acetylhistidine (VIP = 1.976, *P* < 0.001; VIP = 3.406, *P* < 0.001), N-Acryloylglycine (VIP = 1.195, *P* = 0.003; VIP = 1.546, *P* = 0.042), N-Acetyl-L-phenylalanine (VIP = 2.634, *P* < 0.001; VIP = 2.197, *P* = 0.011), and Vinylacetylglycine (VIP = 2.260, *P* < 0.001; VIP = 3.248, *P* = 0.007).

### 3.4 Pathway analysis based on metabolomics

**Figure 3:**
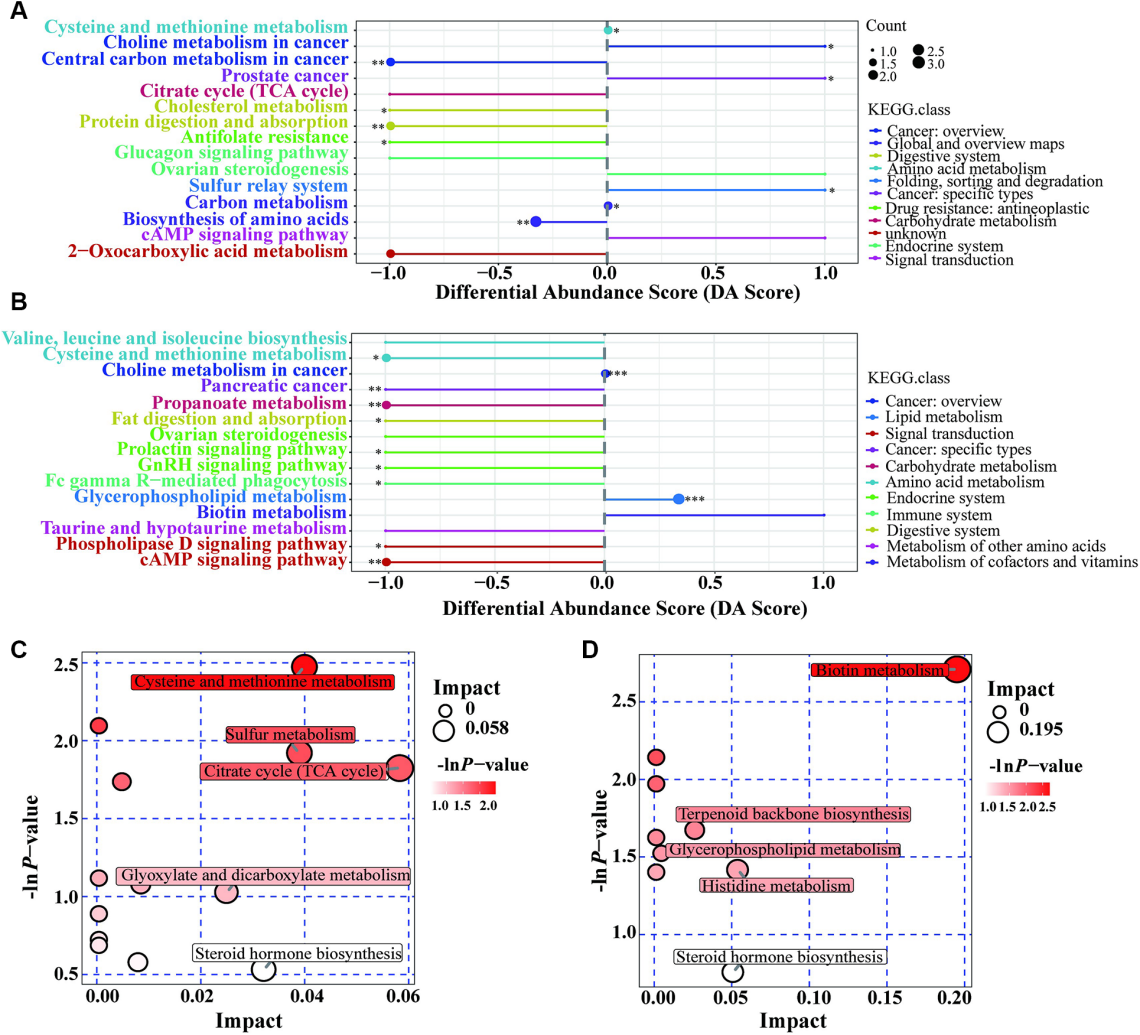
Pathway analysis based on metabolomics. **(A)**: Differential abundance score for Case A vs. Case N; **(B)**: Differential abundance score for Case B vs. Case N; **(C)**: Pathway analysis for g Case A vs. Case N; **(D)**: Pathway analysis for g Case B vs. Case N. DA Score: Ratio of the difference between the number of up-regulated and down-regulated differential metabolites annotated on a pathway to the number of all metabolites on the pathway; Impact: Impact factors obtained through topological analysis.

Pathway enrichment analysis was performed to identify the metabolite pathways associated with altered metabolite levels (Supplementary Table S3). In the Case A group, 10 of the pathways involving the differential metabolites showed significant changes. Figure 3A shows that five pathways were downregulated and three pathways were upregulated. Thus, metabolite disturbances could disrupt the overall metabolic status of GDM patients through metabolic pathways. In the Case B group, 11 pathways showed significant changes, of which only the glycerophospholipid metabolism pathway was upregulated (Figure 3B). Enrichment and topological analysis showed that the critical pathway showing the highest correlation with the differential metabolites was the cycle (TCA cycle) in the Case A group (Figure 3C) and biotin metabolism in the Case B group (Figure 3D). Thus, these may be the main pathways underlying metabolic changes in patients with GDM.

### 3.5 Metabolites with good prediction capacity for neonatal hypoglycemia and macrosomia

**Figure 4:**
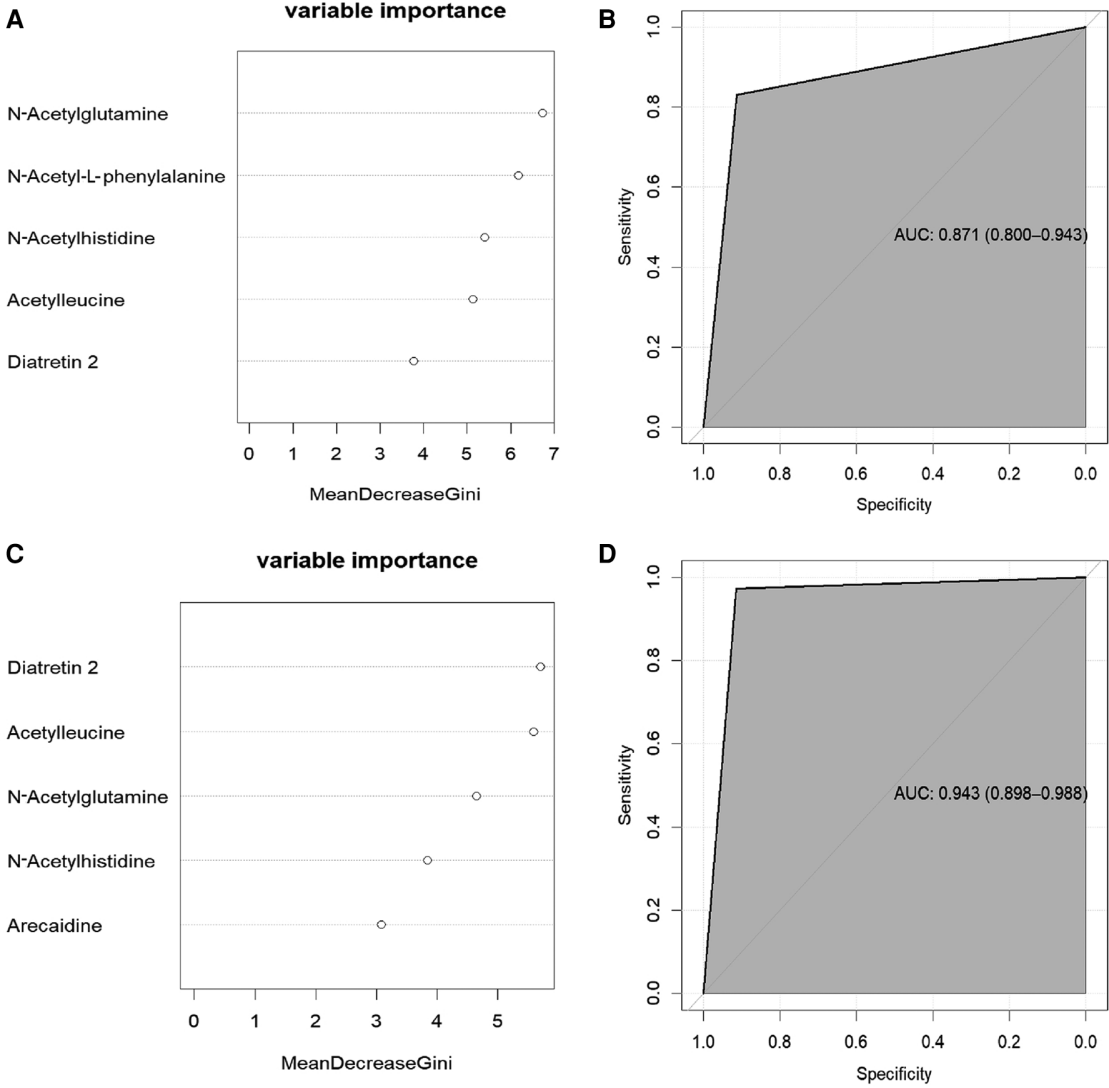
Prediction of adverse offspring outcome in women with GDM. **(A)**: the five metabolites with the greatest reduction in the mean Gini index in the Case A. **(B)**: In the random forest model, the signature with five variables provided the AUC 0.871 for predicting hypoglycaemia. The 95% confidence interval is 0.800-0.943.**(C)**: the five metabolites with the greatest reduction in the mean Gini index in the Case B. **(D)**: In the random forest model, the signature with five variables provided the AUC 0943 for predicting macrosomia. The 95% confidence interval is 0.898-0.988.

The 66 screened serum metabolites for the Case A group and the 14 clinical factors were further characterized using RF analysis. The RF analysis identified *N*-acetylglutamine, *N*-acetyl-l-phenylalanine, *N*-acetylhistidine, acetylleucine, and diatretin 2 as the five metabolites showing the most significant reductions in the mean Gini index (Figure 4A). The datasets of the neonatal hypoglycemia and control groups were randomly divided into training and test sets. An RF-based risk-prediction model using the five metabolites listed above was constructed; the model's error rate was minimized when mtry = 6 and was stabilized when ntree = 800. The RF model achieved the lowest error rate at mtry = 6 and ntree = 800, and showed an AUC of 0.8712 in the test set (Figure 4B), indicating that the selected serum metabolites had good prediction performance.

Similarly, the 45 screened serum metabolites for the Case B group and the 14 clinical factors were also characterized using RF. The five most essential characteristics identified in the RF analysis were diatretin 2, acetylleucine, *N*-acetylglutamine, *N*-acetylhistidine, and arecaidine levels (Figure 4C), all of which were serum metabolite levels. The model achieved the lowest error rate when mtry = 2, and the error rate stabilized when ntree = 700. The AUC for the test set was 0.9434 (Figure 4D), indicating that the model had good prediction accuracy.

These findings indicated that metabolites were suitable biomarkers of neonatal hypoglycemia and macrosomia.

## 4 Discussion

The number of GDM patients has more than doubled since the formulation of the diagnostic criteria for GDM by the IADPSG and their widespread adoption worldwide (27). The criteria formulated by the IADPSG are based on the relationship between hyperglycemia and adverse pregnancy outcomes (28), making them suitable for managing most GDM patients and reducing the incidence of adverse maternal and infant effects. While these standards imply a more rigorous approach to managing the health of patients with GDM, a subset of GDM patients undergoing such management remain at a high risk of adverse infant outcomes such as hypoglycemia and macrosomia, which can combine and seriously threaten the near-and long-term health of the neonate (29, 30). Thus, early identification and appropriate management of high-risk GDM groups are of great practical significance in reducing adverse neonatal outcomes. This study conducted untargeted metabolomics analysis to examine the serum metabolic profile of GDM patients with different pregnancy outcomes. Biomarker panels using a combination of five metabolites in maternal serum obtained at 24–28 weeks of gestation accurately predicted neonatal hypoglycemia or macrosomia among pregnant women with GDM. Generally speaking, our findings could facilitate the identification of high-risk GDM populations with adverse neonatal outcomes. Following the diagnosis of GDM based on the IADPSG criteria, healthcare providers can implement tailored lifestyle interventions including diet and exercise, and the results of our study can make these management more targeted and efficient, which can better protection of maternal and child health.

Previous studies have shown that the incidence of neonatal hypoglycemia and macrosomia in the GDM population was 20%–35% and 4%–27.6%, respectively (30, 31). In our study, the incidence of neonatal hypoglycemia and macrosomia was low, which may be attributable to blood glucose management during pregnancy. Moreover, among the 200 neonates we followed-up, six had both neonatal hypoglycemia and macrosomia, indicating a potential link between these two adverse outcomes. A prospective study indicated that macrosomia is a risk factor for neonatal hypoglycemia (32). Giant fetuses are obviously exposed to higher concentrations of free insulin in the uterus, which can cause a state of metabolic decompensation. As a result, adverse offspring outcomes in women with GDM are expected and may be concurrent.

The metabolic profile of GDM patients with hypoglycemia or macrosomia in their offspring showed significant changes at 24–28 weeks of gestation. Specifically, carbohydrate metabolites 1, 5-anhydroglucitol (1,5-AG), *β*-d-glucosamine, and 1-phosphate fructose were significantly downregulated in the Case A group. Previous studies have proposed that 1,5-AG is sensitive to urinary glucose excretion and can capture glucose variability that cannot be captured by HbA1c measurements (33, 34). Moreover, 1,5-AG has been recently identified included as an essential blood glucose parameter in the study of adverse pregnancy outcomes of diabetes (35, 36). Additionally, our findings indicated that amino acid levels were significantly upregulated in the Case B group, which may be a manifestation of maternal overnutrition. Previous studies have proven that aromatic amino acids, glutamic acid, glutamine, and other amino acids were related to the birth weight, which is also consistent with our research results (37, 38).

In our study, serum metabolites were good predictors of neonatal hypoglycemia and macrosomia. Although previous studies assessed a variety of adverse outcomes as outcome indicators, our study focused on neonatal hypoglycemia and macrosomia, which are more relevant for clinical application, thereby providing a direction for clinical management and prevention and showing more practical significance. In recent years, metabolomics has been increasingly used for the diagnosis and prognostication of GDM, indicating that metabolites play a crucial role in the development and pathogenesis of GDM (39, 40). This approach allows the identification of metabolic observations that can predict poor prognosis in the offspring of GDM patients. A previous study reported that the C-statistic for predicting GDM-related adverse pregnancy outcomes on the basis of social and demographic factors, obstetric and family history, and physical characteristics was less than 0.7 (41). However, in our study, the RF prediction model showed that the top five factors affecting hypoglycemia and macrosomia outcomes were all serum metabolites, and that a prediction model consisting of these five metabolites had AUCs of 0.8712 and 0.9434, respectively, which were superior to the AUCs of clinical indicators in predicting adverse pregnancy outcomes in the offspring of patients with GDM. The use of serum metabolites from weeks 24 to 28 of pregnancy to predict the likelihood of neonatal hypoglycemia and macrosomia in cases of GDM could assist healthcare professionals in implementing proactive measures to prevent these adverse outcomes. Since wearable electrochemical biosensors can be used to monitor metabolites and nutrients (42), in the future, wearable devices that detect serum metabolite levels may be useful for the clinical management of GDM patients to achieve accurate control through early identification of metabolic disorders.

Nevertheless, some limitations of this study require consideration. First, while all participants in this study received consistent diet and exercise counseling, constraints in follow-up prevented us from conducting detailed subject-specific studies of diet and exercise. Therefore, we could not assess the effect of diet and exercise on metabolism during pregnancy. Second, the small sample size of this study may have precluded the evaluation of the differential levels of some metabolites. Metabolites, especially those released at 24–28 weeks of gestation, need to be tested in larger, more diverse populations to assess their predictive value. Third, the range of samples collected in this study was limited, and more case specimens are needed for external verification. Therefore, the results should be interpreted with caution and the predictive potential of the prediction models requires further validation in additional large-sample prospective clinical studies. Last, the metabolomics measurement technique (HPLC-MS) used in this study did not allow for absolute quantitative analysis.

## 5 Conclusions

In summary, we profiled the serum metabolite composition in the context of GDM and further used changed metabolites to predict adverse outcomes in offspring. The high sensitivity of serum metabolites plays a vital role in predicting the risk ofadverse neonatal outcomes in GDM patients. It may be an auxiliary monitoring indicator for the management of GDM in the future.

## Data Availability

All data generated or analyzed during this study are included in this published article and its Supplementary Material. Raw data are not publicly available due to ethical restrictions, since they contain information that could compromise the privacy of research participants, but they are available from the corresponding author on reasonable request.
