# Supplementary material for: Metabolic profiles in gestational diabetes mellitus can reveal novel biomarkers for prediction of adverse neonatal outcomes
Source: Front Pediatr. 2024 Aug 21;12:1432113. doi: 10.3389/fped.2024.1432113 (PMC11371726; doi:10.3389/fped.2024.1432113)
Supplement: Supplementary file 1 [file Datasheet1.zip › supplementary materials/supplementary figure S1.docx]

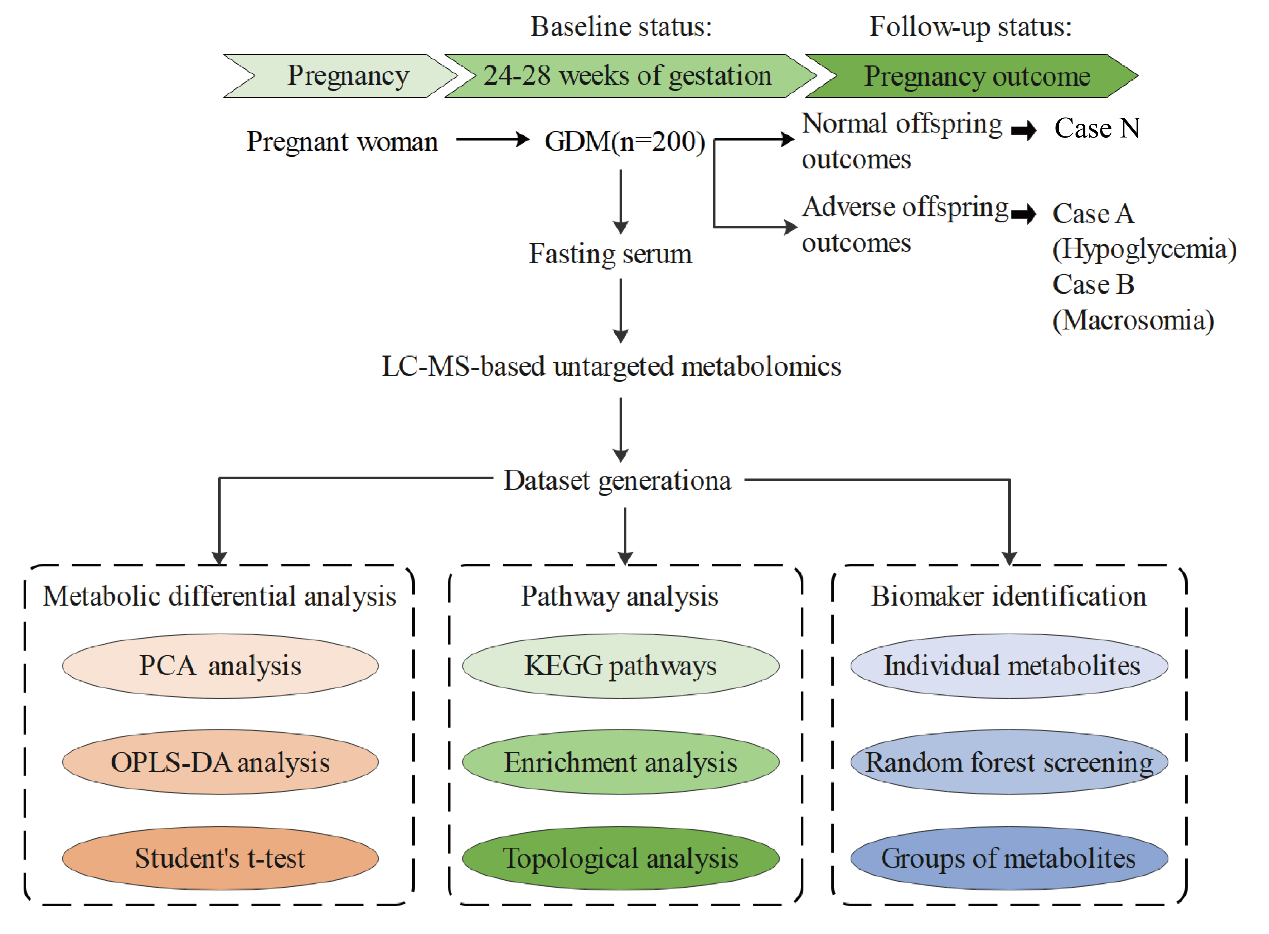


**Supplementary Figure 1. The schematic flow diagram of the study design**. This was a nested case–control study. A total of 200 women were selected out of the participants diagnosed with GDM. Of the 200 selected, 154 women had no adverse outcomes (Case N). 33 women had offspring with hypoglycemia and 19 had macrosomia. This group was termed ‘adverse offspring outcomes’ and it was specifically divided into Case A and Case B group. The fasting serum from the baseline examination was used for LC-MS-based untargeted metabolomics aimed at finding the relation in terms of a predictive signature and the earlier stage pathophysiology of poor offspring outcome of GDM.
